# ABRAXAS1-HELQ is a candidate class-matched reference chimeric RNA and contributes to prostate cell motility through MCOLN2

**DOI:** 10.3389/fonc.2026.1935441

**Published:** 2026-09-28

**Authors:** Jie Chen, Huiwen Tang, Fangdong Li, Jirong Jie, Si Liang, Yiwei Wang, Samir Lalani, Wensen Xiao, Qiong Wang, Qiang Dang

**Affiliations:** 1 Department of Urology, Nanfang Hospital, Southern Medical University, Guangzhou, China; 2 Department of Urology, The Affiliated Traditional Chinese Medicine (TCM) Hospital of Guangzhou Medical University, Guangzhou, China; 3 Department of Pathology, School of Medicine, University of Virginia, Charlottesville, VA, United States

**Keywords:** cell migration, cell motility, chimeric RNA, class-matched reference, prostate

## Abstract

**Background:**

RT-qPCR is widely used for RNA expression quantification, and reliable normalization requires appropriate reference genes. Because different RNA classes display distinct expression characteristics, class-matched internal controls are essential for reliable quantification. While class-matched reference genes have been established for microRNAs and circular RNAs, stable reference genes for chimeric RNAs in the prostate remain lacking, limiting the accuracy of expression analyses. Recent evidence indicates that chimeric RNAs also exist under physiological conditions, suggesting that some chimeric RNAs may function as housekeeping-like molecules.

**Methods:**

RNA sequencing data from the TCGA and CPGEA prostate cancer cohorts were analyzed to identify candidate class-matched reference chimeric RNAs, followed by experimental evaluation in prostate cell lines and prostate tissue samples. Candidate chimeric RNAs were experimentally validated using touchdown PCR, gel electrophoresis, Sanger sequencing, and RT-qPCR. Subcellular localization and biological functions were further evaluated by nuclear-cytoplasmic fractionation, transcriptome analysis, and functional assays.

**Results:**

Analysis of chimeric RNAs shared between TCGA and CPGEA identified 37 candidate chimeric RNAs without a statistically detectable difference between prostate cancer and matched adjacent tissues. ABRAXAS1-HELQ was detectable in the pooled clinical sample, showed a comparatively consistent expression pattern across nine prostate-derived cell lines, and showed no significant change under selected experimental conditions in three cell lines. siRNA-mediated reduction of ABRAXAS1-HELQ was associated with reduced cell migration, with MCOLN2 identified as a potential downstream mediator.

**Conclusion:**

ABRAXAS1-HELQ represents a candidate class-matched reference chimeric RNA with relatively consistent expression under the tested experimental conditions and serves as a potential reference molecule for chimeric RNA quantification in prostate-related studies.

## Introduction

In the 1960s, Nowell and colleagues discovered the Philadelphia chromosome in patients with chronic myeloid leukemia (CML), an abnormality later shown to result from a chromosomal translocation generating the BCR-ABL1 fusion gene (1). This gene encodes the constitutively active tyrosine kinase fusion protein BCR-ABL1, which was established as a key molecular driver of tumorigenesis. Since then, numerous novel chimeric RNAs have been identified across various cancer types, deepening our understanding of oncogenic mechanisms and revealing their potential as therapeutic targets and diagnostic biomarkers. Notable chimeric RNAs include GOLM1-NAA35, which is significantly enriched in esophageal squamous cell carcinoma and correlates with poor progression-free survival (2), and KIF5B-MET, which has been shown to enhance tumor cell proliferation in lung cancer (3). These discoveries contributed to the long-standing perception that chimeric transcripts are predominantly associated with pathological conditions, particularly cancer.

However, advances in sequencing technologies have challenged this long-standing notion. Increasing evidence demonstrates that chimeric RNAs are not confined to pathological states but are also expressed in normal tissues, where they may perform important physiological functions. For example, DUS4L-BCAP29, initially identified in gastric and prostate cancer, was later detected in a variety of normal tissues with identical junction sequences (4, 5). Similarly, CTNNBIP1-CLSTN1 is ubiquitously expressed in normal cells, and its suppression has been shown to significantly impair cell growth and motility (6).

Prostate cancer is one of the most commonly diagnosed malignancies among men worldwide, with approximately 1.5 million new cases and 397,000 deaths estimated in 2022 (7). With the advancement of next-generation sequencing, multiple functionally relevant chimeric RNAs have been discovered in PCa, including TMPRSS2-ERG, which is frequently detected and drives aberrant ERG activation in response to androgen stimulation (8, 9), and SLC45A3-ELK4, a cis-spliced chimeric RNA that promotes cell proliferation primarily in the form of lncRNA (10). Another example is SFT2D2-TBX19, which contributes to PCa progression both by encoding an oncogenic fusion protein and by functioning as a long non-coding RNA that stabilizes mitochondrial ATP synthase via ATP5F1A phosphorylation (11).

Reverse transcription quantitative PCR (RT-qPCR) remains the most widely used technique for measuring RNA expression levels due to its sensitivity and precision. Nevertheless, its accuracy depends heavily on appropriate normalization using reference transcripts whose expression remains sufficiently consistent across the relevant sample types and experimental conditions. Increasing evidence indicates that traditional reference genes vary substantially across tissues (12, 13) and between tumor and adjacent normal samples (14, 15), leading to normalization bias. In addition, different RNA classes display pronounced differences in expression abundance, molecular stability, extraction efficiency, and amplification behavior, making the use of class-matched reference genes particularly important. Accordingly, class-specific reference transcripts have been proposed or commonly used for different RNA types, including GAPDH for mRNAs, miR-16, miR-93, and miR-191 for microRNAs (16), RP11-204K16.1 and XLOC_012542 for lncRNAs (17), and hsa_circ_0000471 and hsa_circ_0000284 for circRNAs (18).

Although numerous functional chimeric RNAs have been identified in prostate cancer, a corresponding class-matched reference RNA for chimeric RNA quantification is still lacking. Because chimeric RNAs are generally expressed at much lower levels than conventional genes, the use of traditional housekeeping genes often leads to mismatched expression ranges and Ct values falling outside the optimal linear interval, thereby compromising quantitative accuracy. Moreover, the instability of commonly used reference genes between tumor and adjacent normal tissues further exacerbates normalization bias. Together, these issues underscore the need to identify candidate class-matched reference chimeric RNAs with sufficiently consistent expression for further evaluation as normalization controls in prostate cancer studies.

In this study, we sought to identify candidate chimeric RNAs showing relatively consistent expression in prostate tissues, investigate their potential biological functions and underlying mechanisms, and preliminarily evaluate their suitability as class-matched reference transcripts for chimeric RNA quantification.

## Materials and methods

### Cell culture

Prostate cancer cell lines 22Rv1, DU145, LNCaP, PC-3, and PC-3M were obtained from Suzhou Haixing Biosciences Co., Ltd. (Suzhou, China). The immortalized benign prostate epithelial cell line BPH-1 was obtained from Procell Life Science & Technology Co., Ltd. (Wuhan, China). The enzalutamide-resistant LNCaP cell line (LNCaP-ENZR), the C4-2 prostate cancer cell line, and HEK293T cells were kindly provided by Prof. Qiang Wei’s laboratory at Guangdong Provincial People’s Hospital. Unless otherwise indicated, cells were cultured in RPMI-1640 medium (Procell, Wuhan, China) supplemented with 10% fetal bovine serum (FBS; HyCyte, Suzhou, China) and 1% penicillin-streptomycin (Beyotime, Shanghai, China) at 37 °C with 5% CO_2_. RWPE-1 cells were obtained from Laibaha Biotechnology (Shanghai, China) and cultured in KM medium under the same conditions. Plasmid transfections were performed using Lipofectamine™ 3000 (Thermo Fisher Scientific, USA).

### Clinical samples

Tumor tissues and matched adjacent normal tissues were obtained from 21 patients with prostate cancer at Nanfang Hospital (Guangzhou, China) for the validation of candidate chimeric RNAs.

### UVC treatment

Prostate cells were cultured in 6-well plates containing 2 mL of complete medium per well until they reached approximately 80% confluence. UVC irradiation was performed using a Philips TUV 30W/G30 T8 ultraviolet lamp (90 cm, Philips, Netherlands), positioned 78 cm above the surface of the culture plates. Cells were exposed to UVC for 0, 0.5, 1, or 2 h. Total RNA was extracted immediately after irradiation.

### Bioinformatics analysis

RNA sequencing (RNA-seq) data and corresponding clinical information were obtained from two major genomic repositories: The Cancer Genome Atlas (TCGA; https://portal.gdc.cancer.gov/) and the Chinese Prostate Cancer Genome Atlas (CPGEA; https://ngdc.cncb.ac.cn/gsa-human/browse/HRA000099). Gene Ontology (GO) and Kyoto Encyclopedia of Genes and Genomes (KEGG) enrichment results were visualized using SangerBox (http://sangerbox.com/). To investigate the potential biological functions of the chimeric RNA, RNA-seq data from the CPGEA cohort were analyzed to identify genes associated with ABRAXAS1-HELQ expression. Samples were stratified into high- and low-expression groups based on the median expression of ABRAXAS1-HELQ. Differentially expressed genes (DEGs) between groups were identified using the limma R package, with |log2FC| > 1 and adjusted P < 0.05 as significance thresholds. Functional annotation of DEGs was conducted by Gene Ontology (GO) and Kyoto Encyclopedia of Genes and Genomes (KEGG) enrichment analyses using the clusterProfiler package.

RNA sequencing was performed in PC-3 and LNCaP cells following ABRAXAS1-HELQ knockdown. Because this analysis was intended for exploratory screening rather than confirmatory differential expression testing, genes meeting the predefined computational filtering criteria of |log2FC| > 1 and an adjusted P value < 0.05 were retained as candidate genes showing exploratory expression changes and were not interpreted as statistically validated differentially expressed genes. Candidate genes showing overlapping expression changes in the two cell lines were identified, and exploratory Gene Ontology (GO) functional enrichment analysis was conducted using GOrilla (https://cbl-gorilla.cs.technion.ac.il/). Detailed information regarding the experimental design, sequencing procedures, and bioinformatic analysis is provided in the **Supplementary Materials and Methods**.

### Lentiviral packaging and stable cell generation

The MCOLN2 lentiviral expression plasmid was constructed by Youming Biotechnology Co., Ltd. (China). For lentivirus production, HEK293T cells were co-transfected with the MCOLN2 plasmid, the packaging plasmid psPAX2, and the envelope plasmid pMD2.G using Lipofectamine™ 3000 (Thermo Fisher Scientific, USA). After 48 h, the viral supernatant was collected, and the viral particles were concentrated by centrifugation and resuspended in phosphate-buffered saline (PBS). The resulting viral particles were used to infect prostate cells seeded in 6-well plates in the presence of polybrene (5 µg/mL). Forty-eight hours after infection, cells were subjected to puromycin selection (2 µg/mL) to establish stable MCOLN2-expressing cell lines.

### Transient transfection

Small interfering RNA (siRNA) oligonucleotides were synthesized by GenePharma (Suzhou, China), and their sequences are provided in **Supplementary Table S2**. The siRNA targeting the chimeric RNA was designed to directly span the chimeric junction, such that its target-complementary sequence contained nucleotides derived from both parental genes. Cells were transiently transfected with the indicated siRNAs using Lipofectamine™ 3000 reagent (Thermo Fisher Scientific, USA) according to the manufacturer’s instructions. After 24-48 h of transfection, cells were harvested for RNA extraction.

### RNA extraction and RT-qPCR

Total RNA was extracted using the EZ-press RNA Purification Kit (EZBioscience, Rosemead, USA) according to the manufacturer’s instructions. One microgram of RNA was reverse transcribed using HiScript^®^ II Q RT SuperMix (Vazyme Biotech). RT-qPCR was performed with ChamQ SYBR qPCR Master Mix (Low ROX Premixed, Vazyme Biotech) on a QuantStudio 6 Flex system (Thermo Fisher Scientific). Gene expression levels were normalized to GAPDH, and relative mRNA levels were calculated using the 2^−ΔΔCt^ method. All primers were synthesized by BGI (Shenzhen, China); sequences are provided in **Supplementary Table S1**.

### Touchdown PCR, agarose gel electrophoresis, and Sanger sequencing

Total RNA (1 µg) was reverse transcribed using the HiScript^®^ III 1st Strand cDNA Synthesis Kit with gDNA wiper (Vazyme Biotech, China) according to the manufacturer’s instructions. The resulting cDNA was used as a template for touchdown PCR (TD-PCR) with 2× Phanta Flash Master Mix (Dye Plus, Vazyme Biotech, China). PCR products were separated by electrophoresis on agarose gels of appropriate concentrations (1.0% for fragments >1 kb; 1.5-2.0% for fragments <1 kb) based on expected amplicon size. Gels were visualized under UV illumination using a gel documentation system (Tanon, China), and target bands were excised and purified using a gel extraction kit. Purified PCR products were subjected to Sanger sequencing by RiboBio Co., Ltd. (Guangzhou, China).

### Nuclear and cytoplasmic RNA isolation

Nuclear and cytoplasmic fractions were prepared using the NE-PER™ Nuclear and Cytoplasmic Extraction Reagents Kit (Thermo Fisher Scientific, USA) according to the manufacturer’s instructions. Total RNA from each fraction was isolated using the EZ-press RNA Purification Kit (EZBioscience, Rosemead, USA).

### CCK-8 assay and colony formation assay

Cell proliferation was assessed using the Cell Counting Kit-8 (CCK-8, APExBIO, USA). Cells were seeded into 96-well plates at a density of 2000 cells per well. At the indicated time points, CCK-8 solution was added according to the manufacturer’s instructions, and absorbance at 450 nm was measured after incubation at 37 °C. For the colony formation assay, prostate cells were seeded into 6-well plates at a density of 500 cells per well and cultured for 10 days. The colonies were then fixed with 4% paraformaldehyde, stained with crystal violet, and colonies containing more than 50 cells were counted.

### Flow cytometric analysis of apoptosis

Cell apoptosis was assessed by Annexin V-FITC/propidium iodide (PI) staining followed by flow cytometry. RWPE-1, BPH-1, PC-3, and LNCaP cells were transfected with negative-control siRNA (siNC) or ABRAXAS1-HELQ junction-targeting siRNA (siA-H). At 72 h after transfection, both adherent and floating cells were collected, washed twice with cold PBS, and resuspended in binding buffer. Cells were subsequently stained with Annexin V-FITC and PI according to the manufacturer’s instructions and incubated in the dark at room temperature for the indicated period. Samples were then analyzed by flow cytometry. Annexin V-positive/PI-negative cells were considered early apoptotic cells, whereas Annexin V-positive/PI-positive cells were considered late apoptotic or secondary necrotic cells. The total apoptotic cell population was calculated as the sum of early and late apoptotic cells. Data were obtained from three independent biological experiments.

### Wound healing and transwell migration

For the wound healing assay, cells were plated in 6-well plates and cultured until approximately 90% confluence. A linear scratch was created across the cell monolayer using a 200 μL pipette tip. After washing twice with PBS to remove detached cells, serum-free medium was added, and images were captured at 0 h and 24 h using an inverted microscope.

Transwell migration assays were performed using 8 μm pore inserts (Corning, USA). PC-3, BPH-1, or RWPE-1 cells (3 × 10^4^) or LNCaP cells (5 × 10^4^) in 200 μL serum-free RPMI-1640 were seeded into the upper chamber, and 800 μL complete medium was added to the lower chamber as a chemoattractant. After 48 h of incubation, cells that migrated to the lower surface of the membrane were fixed with 4% paraformaldehyde, stained with crystal violet, and counted under an inverted microscope (IX71, Olympus, Japan).

### Statistical analysis

Quantitative data are presented as the mean ± standard deviation (SD) unless otherwise stated and were analyzed using GraphPad Prism (version 9.0, GraphPad Software, USA). Comparisons between two groups were performed using paired or unpaired two-tailed Student’s t-tests, as appropriate. Multiple-group comparisons were analyzed using ordinary or repeated-measures one-way ANOVA followed by Tukey’s multiple-comparisons test. Time-course data were analyzed using two-way ANOVA followed by Šídák’s multiple-comparisons test. Unless otherwise stated, experiments were performed using three independent biological replicates (n = 3), and technical replicates were not treated as independent samples for statistical analysis. All statistical tests were two-sided, and *P* < 0.05 was considered statistically significant. Detailed statistical analyses for individual figures are provided in the **Supplementary Materials and Methods**.

## Results

### Identification and preliminary evaluation of ABRAXAS1-HELQ as a candidate class-matched reference chimeric RNA

To systematically identify chimeric RNAs with relatively consistent expression in prostate tissue, we conducted a comprehensive analysis of prostate cancer transcriptome data from the TCGA and CPGEA cohorts, as detailed in a previous study. Candidate chimeric RNAs were then predicted using the EricScript algorithm in TCGA and CPGEA, yielding 25,944 and 101,973 candidates, respectively. Chimeric RNAs were classified into four subtypes based on parental gene splice sites: E/E type, in which both splice junctions coincide with known exon boundaries; E/M or M/E type, in which one junction lies at a known exon boundary and the other within the exon body; and M/M type, in which both junctions lie within exon bodies. Given the lower validation rate of M/M-type transcripts, they were excluded from subsequent analyses. Additionally, chimeric RNAs present in fewer than five individuals were filtered out to improve candidate reliability. Following further validation of junction specificity using the BLAT tool from UCSC Genome Browser, 384 and 1,351 chimeric RNAs were identified in TCGA and CPGEA, respectively (**Figure 1A**). Comparison between the two cohorts revealed 270 TCGA-specific, 1,237 CPGEA-specific, and 114 shared chimeric RNAs (**Figure 1B**) (19). A total of 37 chimeric RNAs showed no statistically significant difference in expression between tumor and adjacent non-tumor tissues.ABRAXAS1-HELQ was among these candidates (**Figure 1C**).

**Figure 1:**
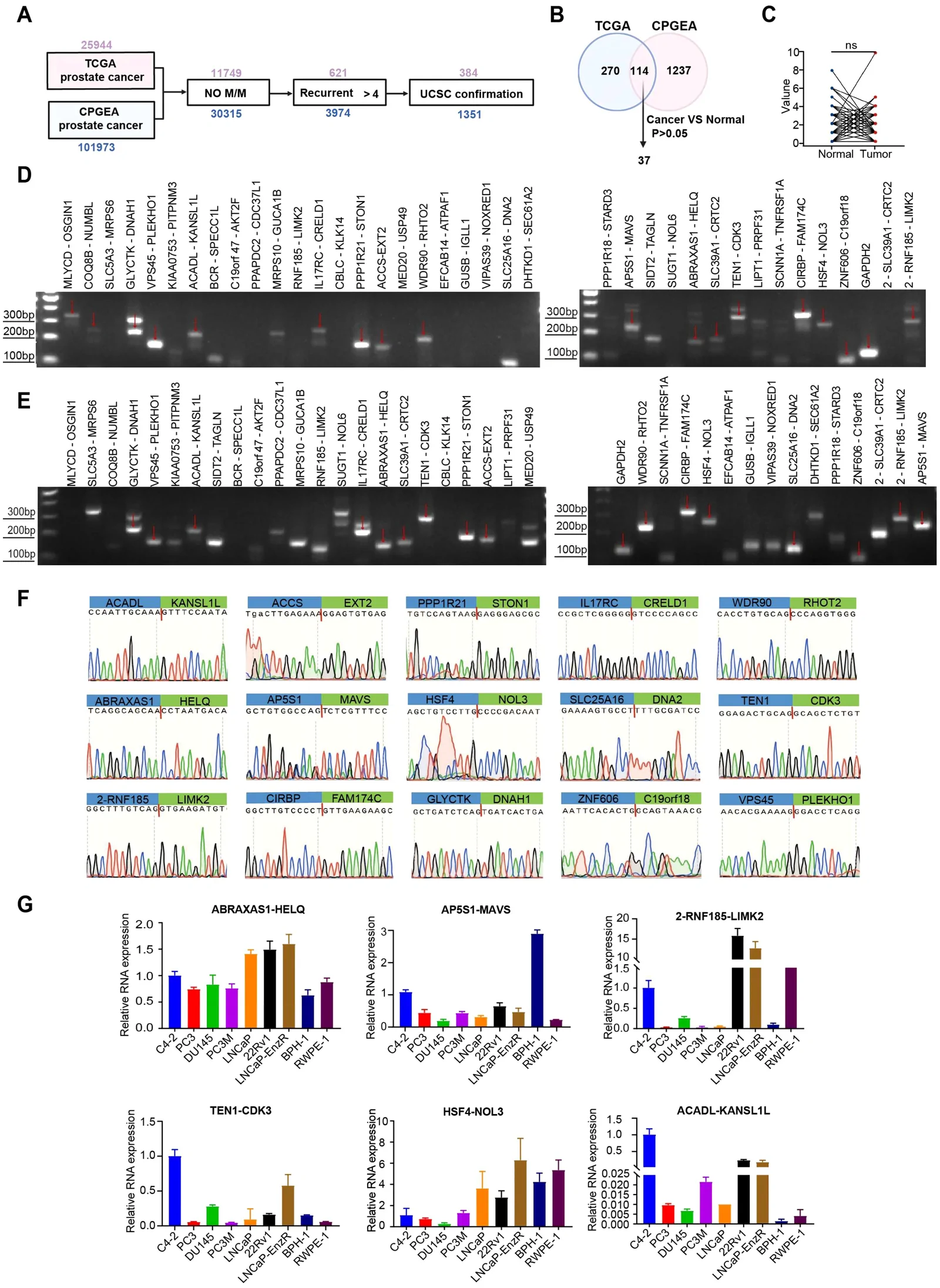
Identification of ABRAXAS1-HELQ as a candidate class-matched reference chimeric RNA. **(A)** Identification and distribution of chimeric RNAs in Western and Chinese PCa cohorts. Transcriptomic data from TCGA (Western) and CPGEA (Chinese) cohorts were analyzed. Following the exclusion of “M/M” fusions and low-recurrence transcripts (n < 5), 384 and 1,351 chimeric RNAs were identified in TCGA and CPGEA, respectively, after UCSC Genome Browser validation. **(B)** Venn diagram showing the overlap of identified chimeric RNAs between the TCGA and CPGEA cohorts. **(C)** Comparative analysis of ABRAXAS1-HELQ expression levels between prostate cancer tissues and adjacent non-tumor tissues using the CPGEA dataset. Statistical significance was assessed using a paired two-tailed Student’s t-test. **(D, E)** Gel electrophoresis of RT-qPCR products for 37 candidate chimeric RNAs in **(D)** the pooled clinical RNA sample and **(E)** the pooled prostate-derived cell-line RNA sample. Red arrows indicate the expected bands. GAPDH was included as an internal control. **(F)** Sanger sequencing confirming that the junction sequences of 15 candidate chimeric RNAs were concordant with the predicted sequences. **(G)** RT-qPCR analysis of the relative expression levels of 15 candidates in nine individual prostate-derived cell lines. Only six representative chimeric RNAs are shown. Data are presented as mean ± SD from three independent biological replicates (n = 3).

Junction-specific primers were designed based on the chimeric RNA breakpoint coordinates. RNA extracted from all 21 prostate cancer tissues and all 21 matched adjacent tissues was combined into a single pooled clinical RNA sample. Junction-specific RT-qPCR was then performed using this pooled RNA sample, and gel electrophoresis of the amplification products detected 18 candidate chimeric RNAs (**Figure 1D**). This analysis confirmed their detectability in the pooled clinical RNA sample but did not assess their expression in individual patients or interpatient expression variability. A separate pooled RNA sample was prepared from nine prostate-derived cell lines, including seven prostate cancer cell lines (22Rv1, PC-3, C4-2, PC-3M, LNCaP-ENZR, LNCaP, and DU145) and two immortalized benign prostate epithelial cell lines (BPH-1 and RWPE-1). RT-qPCR was then performed using this pooled RNA sample, followed by gel electrophoresis of the amplification products. Sixteen candidate chimeric RNAs overlapping with those detected in the prostate tissue screening were identified. In **Figure 1D**, red arrows indicate bands corresponding to the expected product sizes, whereas in **Figure 1E**, red arrows indicate the 16 overlapping candidate chimeric RNAs.

Comparison of the tissue- and cell-line screening results identified 16 candidates that were detectable in both sample types. Sanger sequencing confirmed that the junction sequences of 15 corresponding amplicons matched the predicted sequences (**Figure 1F**). Housekeeping genes are typically expressed at high and stable levels and play essential roles in fundamental cellular processes (20), such as GAPDH in glycolysis, β-actin in cytoskeletal organization and cell motility, and tubulin in microtubule assembly. RT-qPCR analysis of the 15 sequence-confirmed candidates showed that ABRAXAS1-HELQ had a comparatively consistent expression pattern across the nine tested prostate-derived cell lines (**Figure 1G** and **Supplementary Figure S5**). Further analysis using the Cancer Cell Line Encyclopedia (CCLE) analysis showed broad detectability and relatively consistent expression of ABRAXAS1-HELQ across multiple tumor cell lines of different tissue origins (**Figure 2A**), supporting its further evaluation as a candidate class-matched reference chimeric RNA.

**Figure 2:**
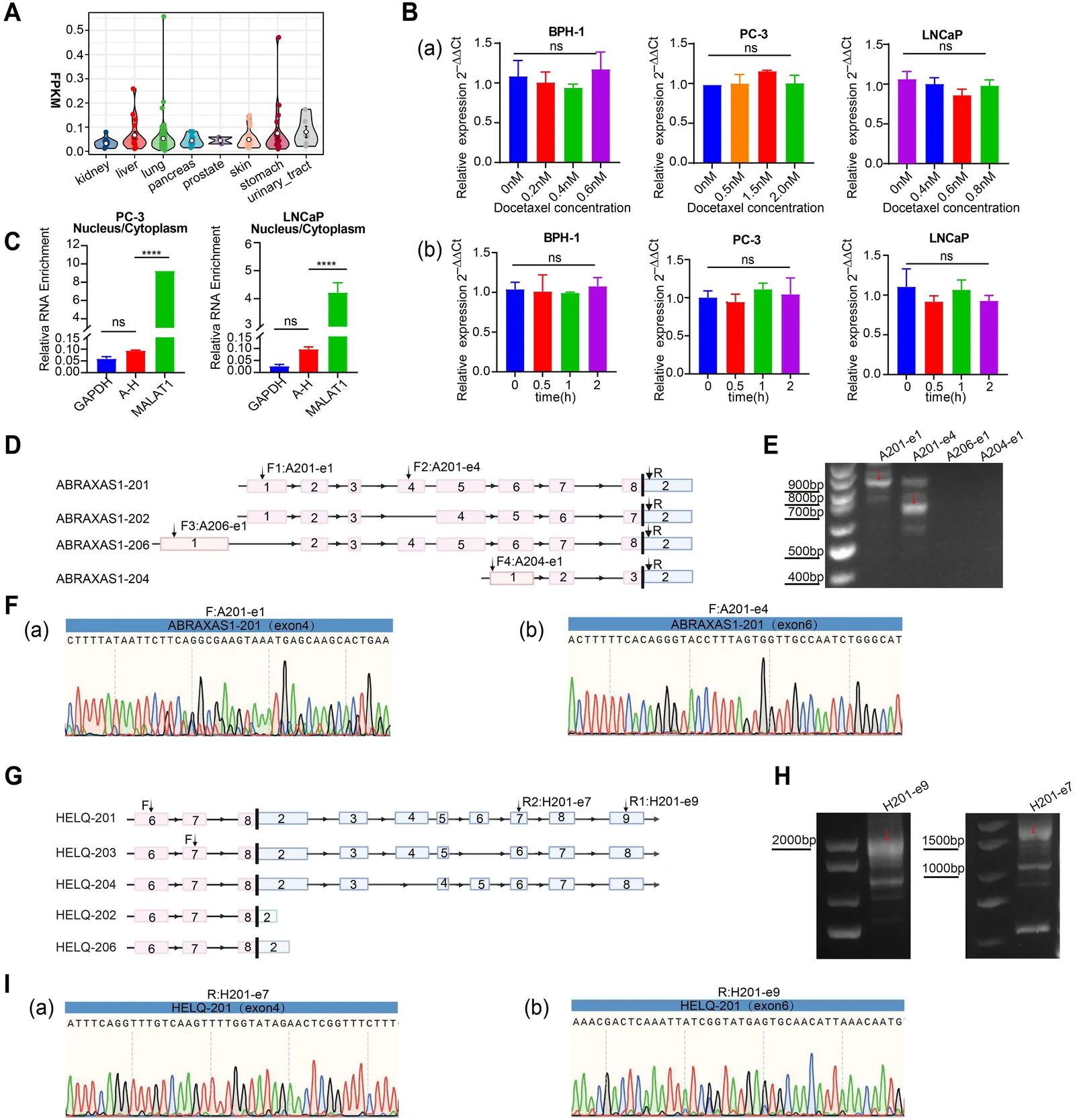
Characterization and transcript-structure validation of chimeric ABRAXAS1-HELQ. **(A)** CCLE database analysis showing the FPKM-based expression levels of ABRAXAS1-HELQ across various tumor cell lines of different tissue origins. **(B)** RT-qPCR analysis of ABRAXAS1-HELQ expression under docetaxel treatment and UVC irradiation. ABRAXAS1-HELQ expression levels were analyzed in PC-3, LNCaP, and BPH-1 cells following: **(a)** 48 h treatment with indicated concentrations of docetaxel; **(b)** exposure to a 30-W UVC source at 254 nm for 0, 0.5, 1, or 2 h. Data are presented as mean ± SD from three independent biological replicates (n = 3). Statistical significance was assessed using ordinary one-way ANOVA followed by Tukey’s multiple-comparisons test. **(C)** Subcellular localization of ABRAXAS1-HELQ in prostate cells. Nucleocytoplasmic fractionation followed by RT-qPCR was performed to determine the relative distribution of ABRAXAS1-HELQ in PC-3 and LNCaP cells. GAPDH and MALAT1 served as cytoplasmic and nuclear markers, respectively. Data are presented as mean ± SD from three independent biological replicates (n = 3). Statistical significance was assessed using one-way repeated-measures ANOVA followed by Tukey’s multiple-comparisons test. **(D, G)** Schematic of primer binding sites targeting splice variants of **(D)** ABRAXAS1 and **(G)** HELQ. **(E, H)** Gel electrophoresis of touchdown PCR products; red arrows indicate the expected amplicons. **(F, I)** Sanger sequencing confirming the presence of exons 4 and 6 in ABRAXAS1-201 **(F)** and exons 4 and 6 in HELQ-201 **(I)**. **** P < 0.0001; ns, P > 0.05.

We next examined whether ABRAXAS1-HELQ expression remained relatively consistent under selected experimental conditions. PC-3, LNCaP, and BPH-1 cells were treated with docetaxel (DTX), a semisynthetic taxane that binds to β-tubulin and stabilizes microtubules, thereby inducing cell-cycle arrest and apoptosis (21), or exposed to UVC (254 nm), which directly damages DNA and is widely used to establish cellular DNA damage models (22–24). RT-qPCR analysis demonstrated that ABRAXAS1-HELQ expression showed no significant changes following DTX treatment or UVC irradiation in PC-3, LNCaP, and BPH-1 cells (**Figure 2B**). These findings indicate relative expression consistency under the tested conditions and support further evaluation of ABRAXAS1-HELQ as a potential class-matched reference chimeric RNA. However, they do not constitute formal validation of its suitability as a quantitative reference. Individual-level clinical validation, broader experimental testing, and formal reference-gene stability analyses are still required before routine use.

### Subcellular localization of the chimeric RNA ABRAXAS1-HELQ

Chimeric RNAs may function as either messenger RNAs (mRNAs) that encode proteins or long noncoding RNAs (lncRNAs) with regulatory roles (10, 25, 26). Protein-coding mRNAs are typically localized in the cytoplasm, whereas long noncoding RNAs are predominantly enriched in the nucleus (27, 28). To determine the subcellular localization of ABRAXAS1-HELQ, nuclear and cytoplasmic fractions were isolated from PC-3 and LNCaP cells, and its expression in each fraction was measured by RT-qPCR. The protein-coding gene GAPDH mRNA and the nuclear lncRNA MALAT1 were used as cytoplasmic and nuclear markers, respectively. The results showed that MALAT1 was mainly enriched in the nucleus, whereas ABRAXAS1-HELQ displayed a distribution pattern similar to that of GAPDH, suggesting that it is primarily localized in the cytoplasm (**Figure 2C**).

### Selected-region validation and parental isoform identification of ABRAXAS1-HELQ

Since ABRAXAS1-HELQ represents a previously unreported chimeric RNA, we sought to validate selected transcript regions and identify the likely parental transcript isoforms contributing to the chimera. We first retrieved its junction sequence using the UCSC Genome Browser. Analysis revealed that the parental gene ABRAXAS1 possesses four splice variants (201, 202, 204, and 206) (**Figure 2D**), whereas HELQ has five variants (201, 202, 203, 204, and 206) (**Figure 2G**).

To examine selected ABRAXAS1-derived transcript regions, splice-variant-specific primers were designed for both parental genes. Touchdown PCR (TD-PCR) was performed using a pooled RNA sample prepared from nine prostate-derived cell lines, including seven prostate cancer cell lines (22Rv1, PC-3, C4-2, PC-3M, LNCaP-ENZR, LNCaP, and DU145) and two immortalized benign prostate epithelial cell lines (BPH-1 and RWPE-1), followed by gel electrophoresis and Sanger sequencing. Primers specific for exon 1 of ABRAXAS1–206 or ABRAXAS1–204 did not generate detectable products, suggesting that these isoforms were absent from the pooled sample or expressed below the detection limit (**Figure 2E**). In contrast, primers targeting exons 1 and 4 of ABRAXAS1-201 successfully amplified the expected fragments, and Sanger sequencing of the A201-e1 and A201-e4 amplicons confirmed the presence of exons 4 and 6, respectively (**Figures 2E, F**). Because ABRAXAS1–202 lacks the region corresponding to exon 4 of ABRAXAS1-201, it was excluded as a potential parental isoform. Together, the exon composition identified within these selected regions supports ABRAXAS1–201 as the most likely ABRAXAS1 parental isoform contributing to ABRAXAS1-HELQ.

To identify the HELQ transcript contributing to the chimeric RNA, specific primers targeting exons 7 and 9 of HELQ-201 were designed for TD-PCR amplification (**Figure 2G**). PCR using HELQ-201-e7 and HELQ-201-e9 primers yielded bands of the expected sizes (**Figure 2H**). Subsequent sequencing revealed that the HELQ-201-e7 amplicon contained exon 4, while the HELQ-201-e9 amplicon included exon 6 (**Figure 2I**). As HELQ-202 and HELQ-206 lack exons 4 and 6, they were excluded as potential parental transcripts. The detection of exon 4 further excluded HELQ-204, and the presence of exon 6 ruled out HELQ-203. Collectively, the exon composition identified within these selected regions supports HELQ-201 as the most likely HELQ parental isoform contributing to ABRAXAS1-HELQ.

### Knockdown of ABRAXAS1-HELQ suppresses prostate cell motility

To explore the biological function of the chimeric RNA ABRAXAS1-HELQ, systematic Gene Ontology (GO) and Kyoto Encyclopedia of Genes and Genomes (KEGG) enrichment analyses were performed using the CPGEA database, and the results were visualized as bubble plots. Among the top seven enriched GO biological process terms, several were related to cell adhesion, suggesting a potential link to cell movement (**Figure 3A**). The top seven enriched cellular component terms included the external side of the plasma membrane, motile cilium, and synaptic membrane (**Figure 3B**). KEGG analysis also identified enrichment of cell motility-related pathways, including the cell adhesion molecules pathway (**Figure 3C**). Taken together, these findings suggest that ABRAXAS1-HELQ may primarily participate in the regulation of cell motility-associated biological processes.

**Figure 3:**
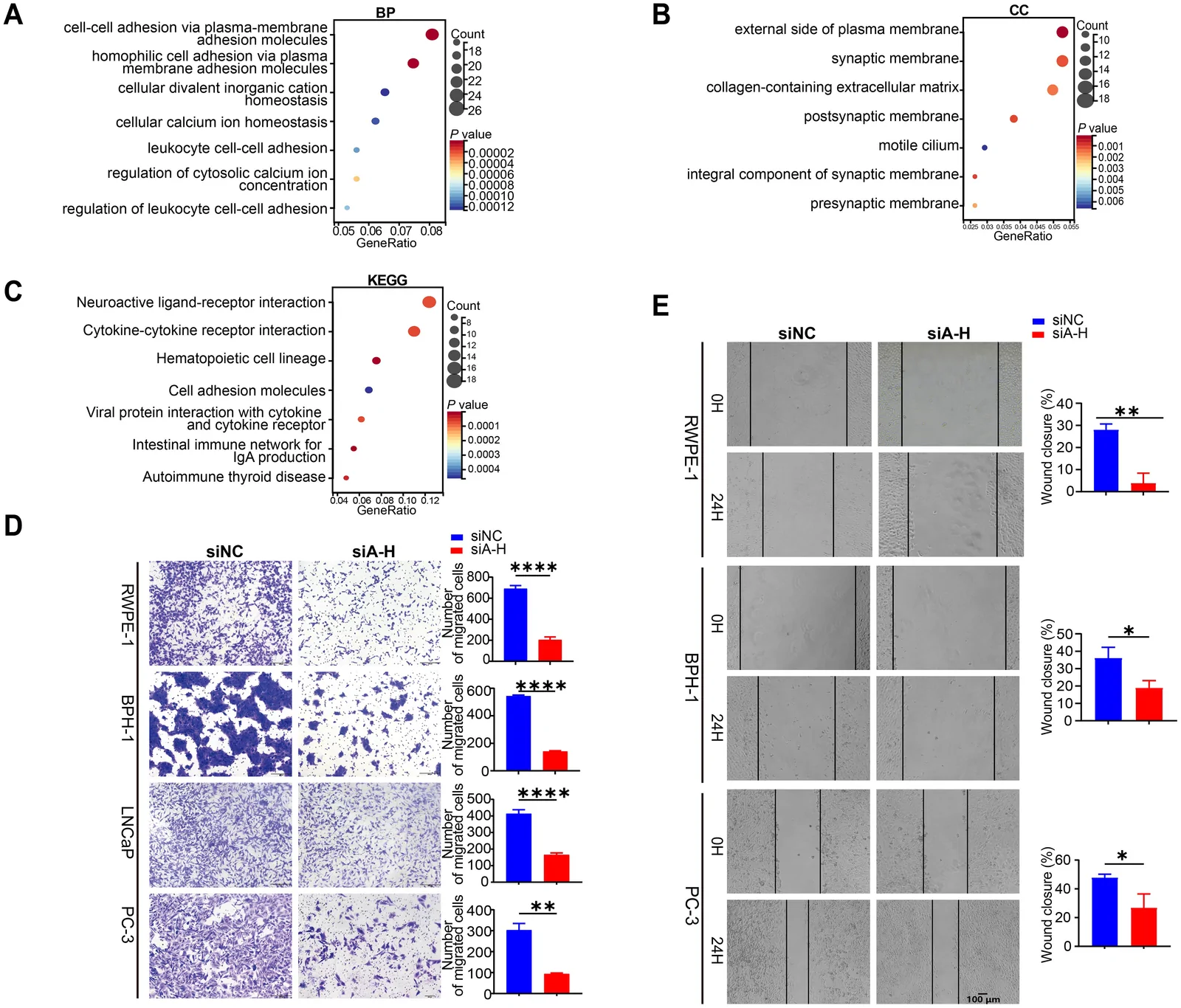
Bioinformatic analysis and functional characterization of ABRAXAS1-HELQ in prostate cells**. (A, B)** Bubble plots illustrating Gene Ontology (GO) enrichment analysis of the ABRAXAS1-HELQ fusion based on the CPGEA database. The top seven enriched terms for **(A)** Biological Process and **(B)** Cellular Component are shown. **(C)** Bubble plot displaying the top seven enriched pathways from Kyoto Encyclopedia of Genes and Genomes (KEGG) analysis. **(D)** Transwell migration assays in RWPE-1, BPH-1, PC-3, and LNCaP cells transfected with negative-control siRNA (siNC) or ABRAXAS1-HELQ junction-targeting siRNA (siA-H). Representative micrographs (left) and corresponding quantification (right) are shown. Scale bars, 200 μm. **(E)** Wound healing assays in RWPE-1, BPH-1, and PC-3 cells transfected with negative-control siRNA (siNC) or ABRAXAS1-HELQ junction-targeting siRNA (siA-H). Representative images at 0 and 24 h and quantification of wound closure are shown. Scale bars, 100 μm. For panels **(D, E)**, quantitative data are presented as mean ± SD from three independent biological replicates (n = 3). Statistical significance was assessed using unpaired two-tailed Student’s t-test for comparisons between siA-H and siNC within each cell line. *P < 0.05, **P < 0.01, ****P < 0.0001; ns, not significant.

To further investigate the involvement of ABRAXAS1-HELQ in cell motility, we transfected both prostate cancer cell lines (PC-3 and LNCaP) and nonmalignant cell lines (BPH-1 and RWPE-1) with an ABRAXAS1-HELQ junction-targeting siRNA (siA-H); efficient knockdown was confirmed by RT-qPCR (**Supplementary Figure S1**). Transwell migration assays in RWPE-1, BPH-1, PC-3, and LNCaP cells showed that siRNA-mediated reduction of ABRAXAS1-HELQ was associated with a marked decrease in the number of migrating cells compared with controls (**Figure 3D**). Similarly, wound-healing assays showed that ABRAXAS1-HELQ knockdown significantly reduced wound closure in PC-3, RWPE-1, and BPH-1 cells compared with controls (**Figure 3E**). Collectively, these concordant observations indicate that siRNA-mediated reduction of ABRAXAS1-HELQ was associated with reduced cell motility in the prostate-derived cell lines tested.

It is noteworthy that inhibition of tumor cell migration does not necessarily equate to suppression of metastatic potential. For instance, β-actin, a key component of the actin cytoskeleton, markedly affects cell motility when downregulated, yet it typically serves as a reference gene rather than a direct regulator of metastasis. We therefore hypothesize that ABRAXAS1-HELQ may function analogously to β-actin.

Because changes in cell proliferation can confound migration-assay readouts, we next examined whether the observed effects were independent of cell proliferation. siRNA knockdown experiments were performed in PC-3, LNCaP, BPH-1, and RWPE-1 cells. CCK-8 assays performed in PC-3, LNCaP, BPH-1, and RWPE-1 cells showed no significant differences in proliferation curves between the siABRAXAS1-HELQ and control groups (**Figure 4A**). Colony-formation assays performed in PC-3, BPH-1, and RWPE-1 cells also revealed no significant differences in colony numbers between the groups (**Figure 4B**). Annexin V-FITC/PI staining followed by flow cytometry revealed no significant differences in the percentages of apoptotic cells between the siA-H and siNC groups in RWPE-1, BPH-1, PC-3, or LNCaP cells (**Figure 4C**). ABRAXAS1-HELQ knockdown did not significantly alter cell proliferation or apoptosis under the conditions tested. These findings suggest that the observed reduction in cell motility is unlikely to be explained by detectable changes in proliferation or apoptosis.

**Figure 4:**
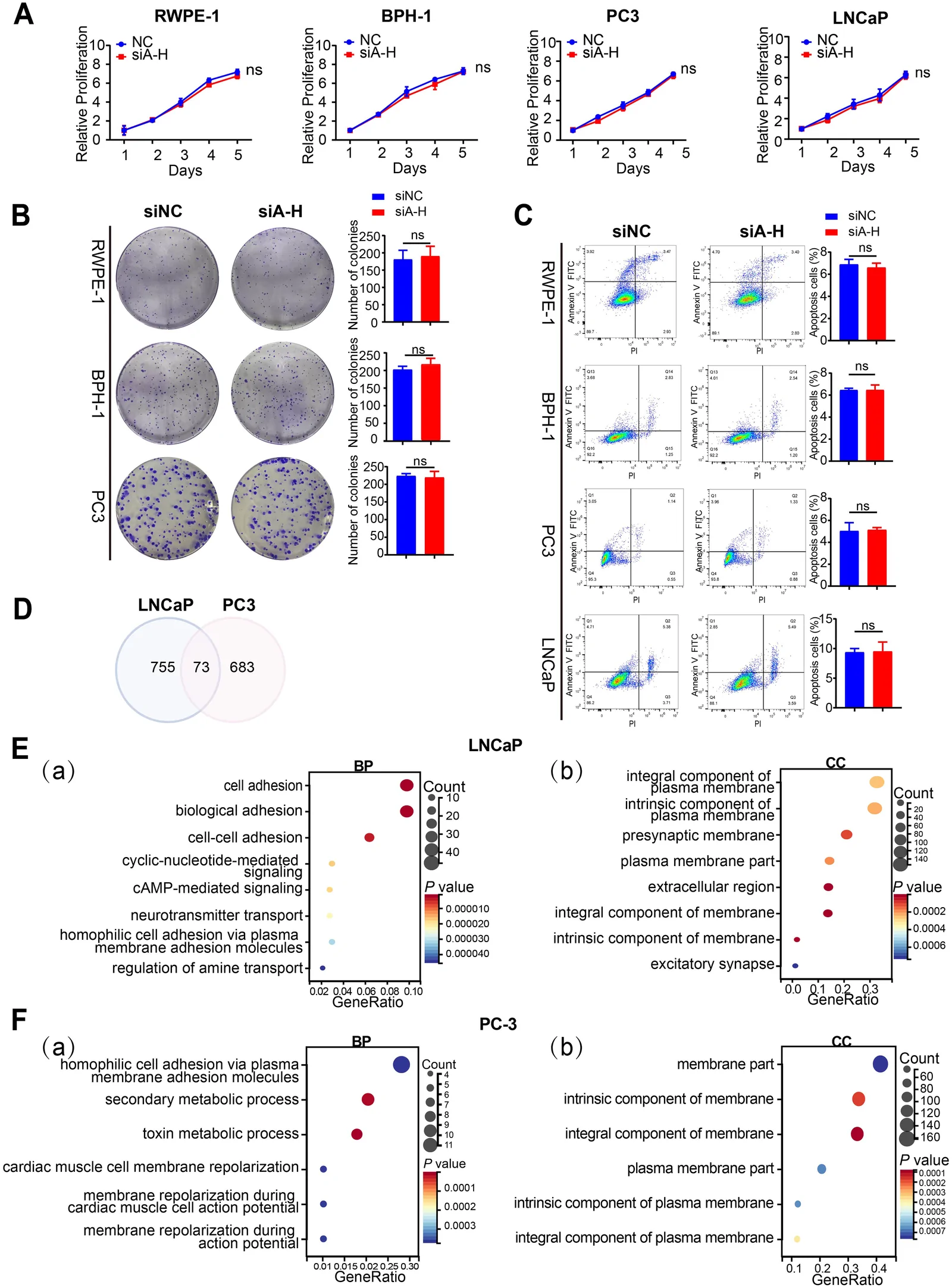
Impact of ABRAXAS1–HELQ knockdown on cell proliferation, apoptosis, and exploratory transcriptomic profiles. **(A)** CCK-8 analysis of RWPE-1, BPH-1, PC-3, and LNCaP cell growth over 5 days after siA-H or siNC transfection. **(B)** Colony formation assays in RWPE-1, BPH-1 and PC-3 cells transfected with siA-H or siNC. Representative images (left) and quantification of colony numbers (right) are shown. **(C)** Flow cytometric analysis of apoptosis in RWPE-1, BPH-1, PC-3, and LNCaP cells 72 h after transfection with siA-H or siNC. Representative plots (left) and quantitative analysis (right) are shown. **(D)** Venn diagram illustrating the distribution of candidate genes retained through exploratory RNA-seq screening in LNCaP and PC-3 cells following ABRAXAS1–HELQ knockdown. The intersection represents 73 candidate genes identified in both cell lines, whereas 755 and 683 candidate genes were identified only in LNCaP and PC-3 cells, respectively. **(E)** Exploratory Gene Ontology **(GO)** analysis of the candidate gene set identified in LNCaP cells. The top eight GO terms returned for **(a)** Biological Process and **(b)** Cellular Component are displayed as bubble plots. **(F)** Exploratory GO analysis of the candidate gene set identified in PC-3 cells. The top six enriched terms for **(a)** Biological Process and **(b)** Cellular Component are displayed as bubble plots. For panels **(A-C)**, quantitative data are presented as mean ± SD from three independent biological experiments (n = 3). For panel **(A)**, statistical significance was assessed using two-way ANOVA followed by Šidák’s multiple-comparisons test for comparisons between siA-H and siNC at each time point. For panels **(B)** and **(C)**, statistical significance was assessed using unpaired two-tailed Student’s t-test for comparisons between siA-H and siNC within each cell line. ns, not significant, P > 0.05.

### ABRAXAS1-HELQ may contribute to prostate cell motility, at least in part, through MCOLN2

To explore potential downstream genes associated with ABRAXAS1–HELQ expression and prostate cell motility, we performed exploratory RNA-seq screening in PC-3 and LNCaP cells following ABRAXAS1–HELQ knockdown. Using the predefined computational screening criteria, 756 and 828 candidate genes showing exploratory expression changes were retained in PC-3 and LNCaP cells, respectively, of which 73 candidate genes were identified in both cell lines (**Figure 4D**). Notably, neither parental ABRAXAS1 nor HELQ was among the candidate genes meeting the exploratory screening criteria in either cell line. Exploratory GO enrichment analysis of these candidate gene sets suggested the overrepresentation of membrane- and cell-adhesion-related terms, including “integral component of membrane,” “intrinsic component of membrane,” “plasma membrane part,” and “cell adhesion” (**Figures 4E, F**), which was consistent with the enrichment patterns observed in the CPGEA dataset.

Given the exploratory nature of the RNA-seq analysis, the 73 candidate genes identified in both cell lines were used only to nominate potential cell-motility-related genes for subsequent independent experimental validation. Among seven candidates analyzed by RT-qPCR, MCOLN2 was the only gene consistently downregulated in both cell lines following ABRAXAS1-HELQ knockdown (**Supplementary Figure S2**). MCOLN2 (Mucolipin TRP Cation Channel 2) belongs to the mucolipin subfamily of the TRP superfamily and encodes a Ca^2+^-permeable, non-selective cation channel implicated in intracellular membrane trafficking and endosomal recycling (29–31). Previous studies have reported that MCOLN2 expression modulates intracellular calcium levels and promotes prostate cell motility (32). Thus, the exploratory RNA-seq analysis served only to nominate MCOLN2 as a potential downstream candidate, whereas the proposed relationship was primarily evaluated through the subsequent RT-qPCR and functional experiments.

To investigate the role of MCOLN2 in cell motility, we transfected BPH-1, RWPE-1, PC-3, and LNCaP cells with siMCOLN2 or negative control siNC, followed by functional assays. RT-qPCR confirmed effective MCOLN2 knockdown (**Supplementary Figure S3**). Transwell migration assays performed in RWPE-1, BPH-1, PC-3, and LNCaP cells, together with wound-healing assays performed in RWPE-1, BPH-1, and PC-3 cells, showed that MCOLN2 knockdown was associated with reduced cell motility compared with the corresponding controls (**Figures 5A, B**).

**Figure 5:**
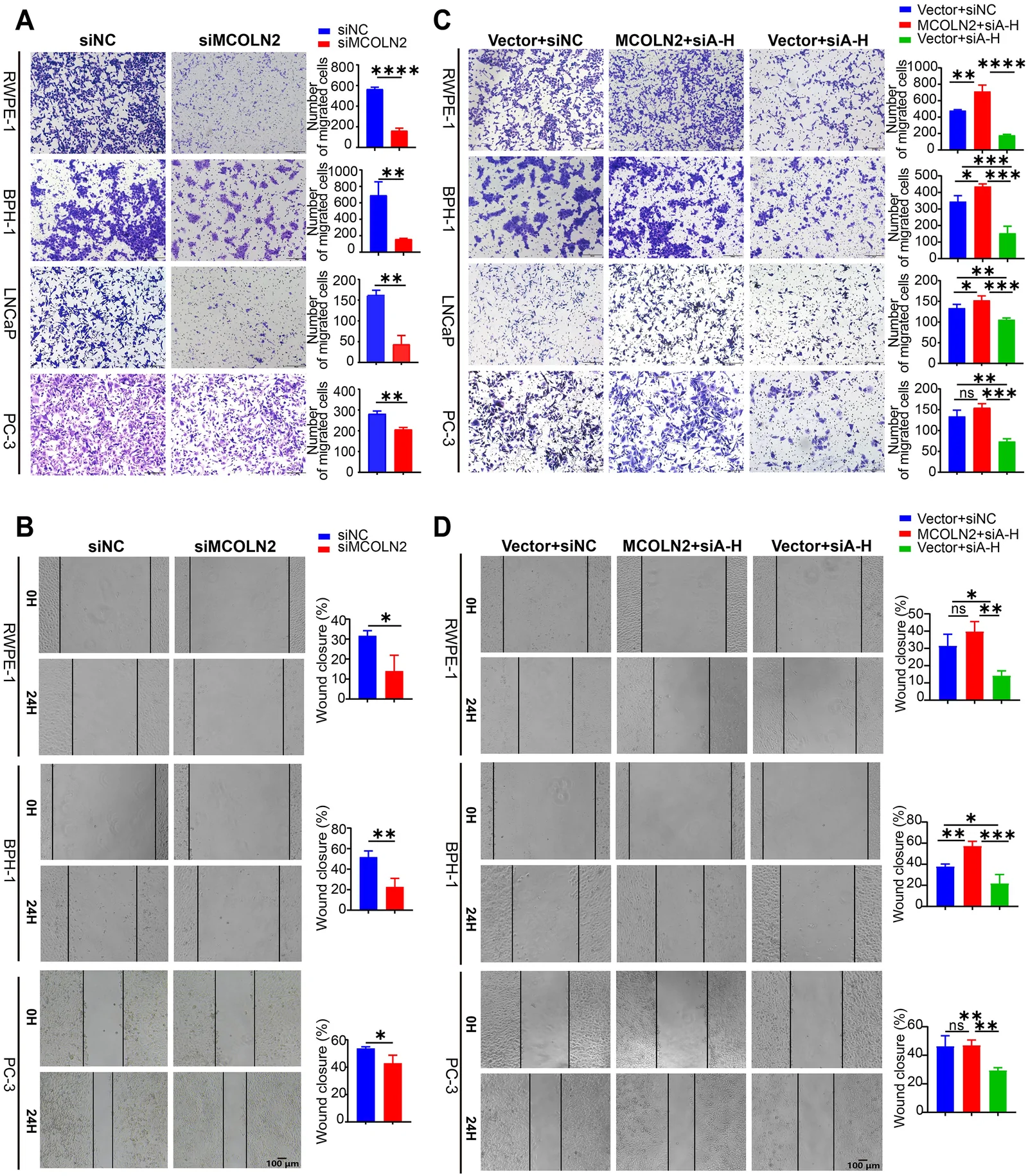
MCOLN2 may contribute to ABRAXAS1-HELQ-related prostate cell motility. **(A)** Transwell migration assays in RWPE-1, BPH-1, PC-3, and LNCaP cells transfected with negative-control siRNA (siNC) or MCOLN2-targeting siRNA (siMCOLN2). Scale bars, 200 μm. **(B)** Wound healing assays in RWPE-1, BPH-1 and PC-3 cells transfected with siNC or siMCOLN2. Representative 0- and 24-h images and wound-closure quantification are shown. Scale bars, 100 μm. **(C)** Transwell migration assays in ABRAXAS1-HELQ-depleted RWPE-1, BPH-1, PC-3, and LNCaP cells with control vector or MCOLN2 re-expression. Scale bars, 200 μm. **(D)** Wound healing assays in ABRAXAS1-HELQ-depleted RWPE-1, BPH-1 and PC-3 cells with control vector or MCOLN2 re-expression. Scale bars, 100 μm. Data are presented as the mean ± SD from three independent biological replicates (n = 3). For panels **(A, B)**, statistical significance was assessed using unpaired two-tailed Student’s t-test for comparisons between siMCOLN2 and siNC within each cell line. For panels **(C, D)**, statistical significance was assessed using ordinary one-way ANOVA followed by Tukey’s multiple-comparisons test for comparisons among the indicated rescue groups within each cell line. *P < 0.05, **P < 0.01, ***P < 0.001, ****P < 0.0001.

Rescue experiments were conducted by re-expressing MCOLN2 in ABRAXAS1-HELQ-depleted BPH-1, RWPE-1, PC-3, and LNCaP cells. RT-qPCR analysis confirmed successful MCOLN2 overexpression (**Supplementary Figure S4**). In RWPE-1, BPH-1, PC-3, and LNCaP cells, Transwell migration assays showed that MCOLN2 re-expression partially restored the migratory capacity reduced following ABRAXAS1-HELQ knockdown (**Figure 5C**). Consistent effects were observed in wound-healing assays performed in PC-3, BPH-1, and RWPE-1 cells (**Figure 5D**). Collectively, the independent RT-qPCR, MCOLN2-knockdown, and rescue experiments provide the principal evidence supporting the possibility that ABRAXAS1–HELQ contributes to prostate cell motility, at least in part, through MCOLN2 (**Figure 6**).

**Figure 6:**
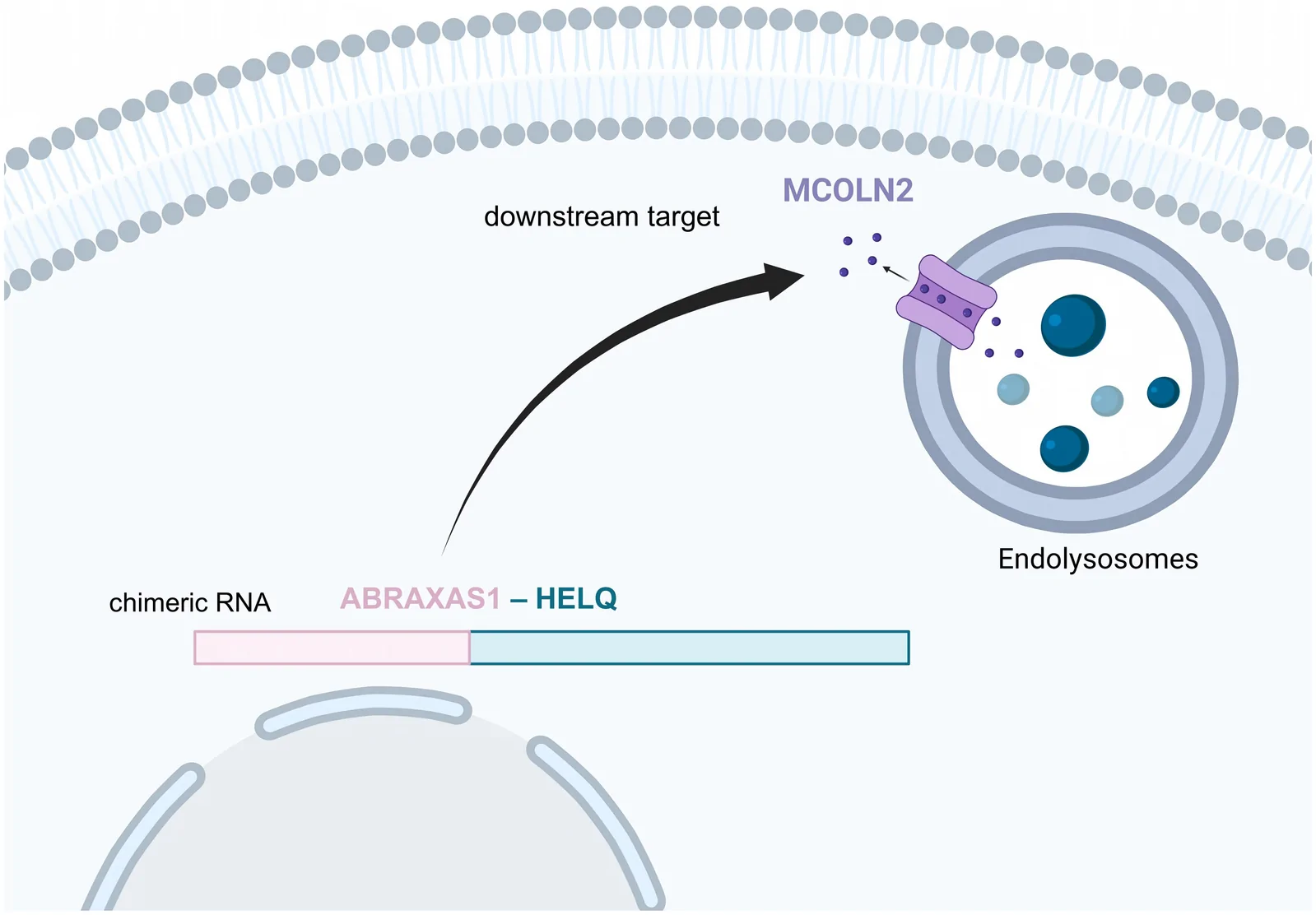
Schematic illustration of the potential functional relationship between ABRAXAS1-HELQ and MCOLN2 in prostate cell motility.

## Discussion

In this study, we identified ABRAXAS1-HELQ as a candidate class-matched reference chimeric RNA for prostate-related research. Integrative analysis of TCGA and CPGEA datasets, together with experimental evaluation in prostate-derived cell lines, showed that ABRAXAS1-HELQ exhibited relatively consistent expression under the tested conditions. CPGEA data further showed no significant difference in ABRAXAS1-HELQ expression between prostate cancer and matched adjacent tissues, whereas pooled clinical samples confirmed its detectability in prostate specimens. Functional assays further revealed that ABRAXAS1-HELQ promotes prostate cell migration, potentially through the regulation of MCOLN2.

Although the biological relevance of chimeric RNAs in normal physiology has received increasing attention, their reliable quantification remains challenging because suitable class-matched reference transcripts are lacking. Although established internal controls are available for other RNA classes, such as microRNAs and circular RNAs, no universally accepted reference chimeric RNA has been established, particularly for prostate-related studies. In this context, our findings identify ABRAXAS1-HELQ as a potential class-matched reference chimeric RNA. Its relatively consistent expression across the tested prostate-derived cell lines and experimental conditions supports its further evaluation as a candidate normalizer for RT-qPCR-based chimeric RNA quantification. However, the current evidence does not establish ABRAXAS1-HELQ as a formally validated or universally applicable internal reference. Individual-sample validation, formal stability analyses, and testing across broader biological and technical conditions will be required before its routine use.

Beyond its methodological contribution, our work also provides insight into the broader biological landscape of chimeric RNAs. Emerging evidence indicates that chimeric RNAs are not restricted to tumors but are also expressed in normal tissues, where they may contribute to essential physiological functions. For instance, DUS4L-BCAP29 has been detected in multiple normal human tissues and non-cancerous epithelial and fibroblast cell lines (33) while JAZF1-JJAZ1 is expressed in both endometrial stromal and tumor tissues (34), suggesting that chimeric RNA formation may be an intrinsic aspect of gene expression rather than merely a pathological event. Evolutionary studies further indicate that chimeric RNA formation and trans-splicing occur across diverse species and may contribute to transcriptomic diversity and adaptation to different physiological conditions (35, 36). These findings suggest an evolutionary link between physiological and pathological RNA processing: malignant transformation may “hijack” normal chimeric RNA machinery to establish aberrant regulatory networks, thereby promoting tumorigenesis. Thus, investigating the functions of physiological chimeric RNAs not only clarifies their roles in maintaining cellular equilibrium but also provides new perspectives for understanding tumor biology and identifying potential therapeutic targets.

Nevertheless, several limitations of our study should be acknowledged. Although 37 candidate chimeric RNAs were initially screened, ABRAXAS1-HELQ only showed the most consistent expression pattern among the candidates examined under the tested conditions. The experimental sample set, which included nine prostate-derived cell lines and a single pooled clinical RNA sample, remained limited in diversity and size, potentially underrepresenting variations due to patient genetics, tumor stage, or treatment history. Therefore, this experiment demonstrated only the detectability of the candidate chimeric RNAs in the combined clinical sample and did not permit evaluation of interpatient variability or experimental comparison between cancer and adjacent tissues. In addition, our PCR and Sanger sequencing experiments validated selected regions of the ABRAXAS1-HELQ transcript and supported identification of ABRAXAS1–201 and HELQ-201 as the likely parental isoforms. However, these data did not establish a continuous 5′-to-3′ sequence of the complete chimeric transcript. Definitive characterization of the complete transcript structure will require approaches such as RACE, full-length cDNA sequencing, or long-read sequencing. An additional limitation is that the functional experiments relied on a single siRNA targeting the ABRAXAS1-HELQ chimeric junction. Although neither parental ABRAXAS1 nor HELQ was included among the candidate genes showing expression changes in the exploratory RNA-seq screening of PC-3 and LNCaP cells, this finding provides only limited support for siRNA specificity, and off-target effects cannot be excluded. Moreover, although our findings support a potential functional relationship between ABRAXAS1-HELQ and MCOLN2, the upstream regulatory mechanisms, direct molecular interactions, and *in vivo* significance remain to be elucidated. Formal stability analyses using NormFinder, geNorm, BestKeeper, or RefFinder, together with validation under broader biological and technical conditions, will also be required before ABRAXAS1-HELQ can be considered for routine use as a quantitative reference. Representative chimeric RNAs were not quantified using ABRAXAS1-HELQ as a normalization control, and the resulting measurements were not compared with those obtained using conventional housekeeping genes or other candidate reference RNAs.

Collectively, our findings identify ABRAXAS1-HELQ as a candidate class-matched reference chimeric RNA that showed relatively consistent expression under the tested conditions and support its further evaluation for prostate-related chimeric RNA quantification. However, individual-level clinical validation, testing under broader biological and technical conditions, and formal reference-gene stability analyses are required before its routine use as a quantitative reference. Our results also suggest that ABRAXAS1-HELQ contributes to prostate cell motility, at least in part, through MCOLN2, providing a basis for further investigation of the methodological and biological relevance of chimeric RNAs.

## Conclusion

We identified ABRAXAS1-HELQ as a candidate class-matched reference chimeric RNA with relatively consistent expression across the tested prostate-derived cell lines and experimental conditions. Functional analyses further suggest that ABRAXAS1-HELQ contributes to prostate cell motility, at least in part, through MCOLN2. These findings support the further evaluation of ABRAXAS1-HELQ as a potential reference for chimeric RNA quantification in prostate-related research.

## Data Availability

The raw RNA-seq data generated in this study have been deposited in the NCBI Sequence Read Archive (SRA) under the BioProject accession number PRJNA1406160.
