# Supplementary material for: ABRAXAS1-HELQ is a candidate class-matched reference chimeric RNA and contributes to prostate cell motility through MCOLN2
Source: Front Oncol. 2026 Sep 28;16:1935441. doi: 10.3389/fonc.2026.1935441 (PMC13620418; doi:10.3389/fonc.2026.1935441)
Supplement: Supplementary file 1 [file SupplementaryFile1.docx]

**Supplementary Figure S1. Validation of ABRAXAS1-HELQ knockdown efficiency.** Relative mRNA levels of *ABRAXAS1-HELQ* were determined by RT-qPCR in RWPE-1, BPH-1, PC-3, and LNCaP cells 24 h after transfection with siABRAXAS1-HELQ or siNC. ** *p*<0.01, and *** *p*<0.001.

**Supplementary Figure S2. mRNA levels of seven candidate genes in PC-3 and LNCaP cells after ABRAXAS1-HELQ knockdown.** The mRNA expression of seven candidate genes selected from exploratory RNA-seq analysis was measured by RT-qPCR in PC-3 and LNCaP cells transfected with siA-H or negative control siRNA (siNC). ns, not significant; ***P* < 0.01.

**Supplementary Figure S3. Validation of MCOLN2 knockdown efficiency.** Relative mRNA levels of MCOLN2 were determined by RT-qPCR in RWPE-1, BPH-1, PC-3, and LNCaP cells 24 h after transfection with siMCOLN2 or siNC.

***P* < 0.01; *****P* < 0.0001.

**Supplementary Figure S4. Validation of MCOLN2 overexpression efficiency.** Relative mRNA levels of MCOLN2 were determined by RT-qPCR in RWPE-1, BPH-1, LNCaP, and PC-3 cells 24 h after transfection with empty vector or MCOLN2 overexpression plasmid. The comparison groups were empty vector versus MCOLN2 overexpression plasmid in each cell line. ****P* < 0.001; *****P* < 0.0001.

**Supplementary Figure S5. RT-qPCR analysis of the relative expression levels of 15 candidate chimeric RNAs in nine prostate-derived cell lines.**
