# Supplementary material for: ABRAXAS1-HELQ is a candidate class-matched reference chimeric RNA and contributes to prostate cell motility through MCOLN2
Source: Front Oncol. 2026 Sep 28;16:1935441. doi: 10.3389/fonc.2026.1935441 (PMC13620418; doi:10.3389/fonc.2026.1935441)
Supplement: Supplementary file 2 [file Table1.docx]

**Supplementary Table S1: Primer sequences used for RT-PCR and RT-qPCR in this study**

| **Primer name** | **Sequence (5'–3')** |
| --- | --- |
| MLYCD-OSGIN1V | GCAACATCCAGGCAATCGT(sense) |
|  | ATGGCATCTGGCTTCGTGT(antisense) |
| COQ8B-NUMBL | AGTTTGGGACAGAGTTCACAGAC(sense) |
|  | CTGCCGTAACTTGTTCATGGT(antisense) |
| SLC5A3-MRPS6 | GGCAGAAACACCAGTTGACG(sense) |
|  | TGCTGACTGTGGGCAGAGAT(antisense) |
| GLYCTK-DNAH1 | AAGCCACATAGCCGTGTCCA(sense) |
|  | AGCCGAAGCTCCCTTCAAGA(antisense) |
| VPS45-PLEKHO1 | GCCACCTATGAAGAGGCTCTAA(sense) |
|  | AAAATCCCTTTCCCGCAG(antisense) |
| KIAA0753-PITPNM3 | TGTAGGCTCCTTCAACCCGT(sense) |
|  | CCCTTCTCCTCTGGCATCAA(antisense) |
| ACADL-KANSL1L | GTGTAACCCGAGCATTTGTGG(sense) |
|  | TTTGCTGTTGCCTCCCTCA(antisense) |
| BCR-SPECC1L | GTGATGCTTTTTTGCCTTGG(sense) |
|  | CCTGCTTGCTTTCTTCATTCTG(antisense) |
| C19orf47-AKT2 | CATCATCAAGAGACTGGGCG(sense) |
|  | TTCATGGTGGCAGCGTGGTA(antisense) |
| PPAPDC2-CDC37L1 | TCCCTGAAGATCTGTTGCCAC(sense) |
|  | CCCGTTGCCTCAGTTCTGAT(antisense) |
| MRPS10-GUCA1B | AGCACAGCAGATGTCTACTTGG(sense) |
|  | CAGCCATTGCCATCCTTATC(antisense) |
| RNF185-LIMK2 | CACCATCCTCAGAAGCCAAGT(sense) |
|  | TGCCAGGTTTCGTTGACAGTC(antisense) |
| IL17RC-CRELD1 | GCTATGGGACGATGACTTGG(sense) |
|  | AGATAGGTCCTGGGAGGTTGAG(antisense) |
| CBLC-KLK14 | TGACCTCATCTAACCCACCCCT(sense) |
|  | AATCCCTACCACAGCTCCCATC(antisense) |
| PPP1R21-STON1 | CCAAGAGGAGTTACGAGGATCA(sense) |
|  | CCCCAAAGTTTTTCTGGTGTC(antisense) |
| ACCS-EXT2 | GAAAACCATGCCCGGCTCAA(sense) |
|  | TTCTTGGGATGAGGGCAGGA(antisense) |
| MED20-USP49 | GCTGTTTTACTCCGTGCCTTAC(sense) |
|  | CGCCACTTTCTCAACCCATT(antisense) |
| WDR90-RHOT2 | GCTGTTGGCTTTGCTGAGTG(sense) |
|  | GGCTTCTGAGTAGTCCACGATG(antisense) |
| EFCAB14-ATPAF1 | TTTGATTCCGATGGAGATGG(sense) |
|  | CTTCCGAAATTCACTGCGTT(antisense) |
| GUSB-IGLL1 | AGAAAATACGTGGTTGGAGAGC(sense) |
|  | CGTCAAGATTCCCGGATAAAAG(antisense) |
| VIPAS39-NOXRED1 | TCTGTCCAAGGAACATCATCTG(sense) |
|  | AGGAGTGGCTGAATTAAGGGA(antisense) |
| SLC25A16-DNA2 | CTACCCATTTGATGTGACTCGTC(sense) |
|  | CCTCCCAAAAACTCTTCTCCA(antisense) |
| DHTKD1-SEC61A2 | TTTGCCATCATCCGAGTAGAG(sense) |
|  | AACAGTGGGATCTGACAACACAC(antisense) |
| PPP1R1B-STARD3 | TGGGCAAAAGACAACCTGTG(sense) |
|  | CAGCTTGCTCATCCTGGTG(antisense) |
| AP5S1-MAVS | GCCTTCCTCATTCACACCTTG(sense) |
|  | AACGGCATTGCTGCTCAGA(antisense) |
| SIDT2-TAGLN | CTTTGACGACCACGACATCTG(sense) |
|  | TGCACTATGATCCACTCCACC(antisense) |
| SUGT1-NOL6 | CTGTTGCTGATGCAAAGAAGTC(sense) |
|  | ATTAAGCTCCTCATTGGTAGGC(antisense) |
| ABRAXAS1-HELQ | ATTGAGAAAAGGAGAGGAGCAC(sense) |
|  | TTCAGTGGCAAAGTCTGTAGCA(antisense) |
| SLC39A1-CRTC2 | CCAGGAGCTAACCATGAAGG(sense) |
|  | TTGTGTATGCCAGTCGCAGT(antisense) |
| TEN1-CDK3 | GGTGATGAATCCAGCCCCTT(sense) |
|  | TCCAGCAGTCGGACGATGTT(antisense) |
| LIPT1-PRPF31 | TCGTATGACGCACTTTTCCA(sense) |
|  | TGCAGTTTATGAGGGTTCTGTG(antisense) |
| SCNN1A-TNFRSF1A | TGAGTCTCCCTCTGTCACGATG(sense) |
|  | TCTCTCTTCTCCCTGTCCCCTA(antisense) |
| CIRBP-FAM174C | GGAGTAACGCTGCTTTGCTT(sense) |
|  | CATCTCCTGTCCTTCCCTCAT(antisense) |
| HSF4-NOL3 | CTTCAGCCCCCTCAAGAAA(sense) |
|  | GTGCATCCAATGCCTCGTA(antisense) |
| ZNF606-C19orf18 | AATCCTTCAGTCAGAGCTGTCAC(sense) |
|  | GAACACTTTGGGCTCTTTGGT(antisense) |
| GAPDH | CAAGGCTGAGAACGGGAAG(sense) |
|  | TGAAGACGCCAGTGGACTC(antisense) |
| A201-E1-H201-E2 | CTCAACACGGACTCGGACA(sense) |
|  | TCAGTGGCAAAGTCTGTAGCA(antisense) |
| A201-E4-H201-E2 | GGCGAAGTAAATGAGCAAGC (sense) |
|  | TCAGTGGCAAAGTCTGTAGCA(antisense) |
| A201-E6-H201-E9 | CATGTCTGAACAACTGGGTT (sense) |
|  | AATGGGATAGTGCGCTTT (antisense) |
| A201-E8-H201-E7 | AGAGGAGCACAGATTCAGGC (sense) |
|  | CACTCATACCGATAATTTGAGTCG(antisense) |

**Supplementary Table S2. Sequences of siRNAs used in this study**

| **siRNA** | Sequence (5′–3′) |
| --- | --- |
| siABRAXAS1-HELQ | GCAGCAACCUAAUGACAGUTT (sense)  ACUGUCAUUAGGUUGCUGCTT (antisense) |
| siNC | UUCUCCGAACGUGUCACGUTT(sense)  ACGUGACACGUUCGGAGAATT(antisense) |
| siMCOLN2 | GCUCGAUUGUGUUCAAUUATT(sense)  UAAUUGAACACAAUCGAGCTT(antisense) |
